# A Naturally Occurring *rev1-vpu* Fusion Gene Does Not Confer a Fitness Advantage to HIV-1

**DOI:** 10.1371/journal.pone.0142118

**Published:** 2015-11-10

**Authors:** Simon M. Langer, Kristina Hopfensperger, Shilpa S. Iyer, Edward F. Kreider, Gerald H. Learn, Lan-Hui Lee, Beatrice H. Hahn, Daniel Sauter

**Affiliations:** 1 Institute of Molecular Virology, Ulm University Medical Center, 89081, Ulm, Germany; 2 Departments of Medicine, Perelman School of Medicine, University of Pennsylvania, Philadelphia, Pennsylvania, 19104, United States of America; 3 Department of Laboratory, Kunming Branch, Taipei City Hospital, 10844, Taipei, Taiwan; Florida Atlantic University, UNITED STATES

## Abstract

**Background:**

Pandemic strains of HIV-1 (group M) encode a total of nine structural (*gag*, *pol*, *env*), regulatory (*rev*, *tat*) and accessory (*vif*, *vpr*, *vpu*, *nef*) genes. However, some subtype A and C viruses exhibit an unusual gene arrangement in which the first exon of *rev* (*rev1*) and the *vpu* gene are placed in the same open reading frame. Although this *rev1*-*vpu* gene fusion is present in a considerable fraction of HIV-1 strains, its functional significance is unknown.

**Results:**

Examining infectious molecular clones (IMCs) of HIV-1 that encode the *rev1-vpu* polymorphism, we show that a fusion protein is expressed in infected cells. Due to the splicing pattern of viral mRNA, however, these same IMCs also express a regular Vpu protein, which is produced at much higher levels. To investigate the function of the fusion gene, we characterized isogenic IMC pairs differing only in their ability to express a Rev1-Vpu protein. Analysis in transfected HEK293T and infected CD4+ T cells showed that all of these viruses were equally active in known Vpu functions, such as down-modulation of CD4 or counteraction of tetherin. Furthermore, the polymorphism did not affect Vpu-mediated inhibition of NF-кB activation or Rev-dependent nuclear export of incompletely spliced viral mRNAs. There was also no evidence for enhanced replication of Rev1-Vpu expressing viruses in primary PBMCs or *ex vivo* infected human lymphoid tissues. Finally, the frequency of HIV-1 quasispecies members that encoded a *rev1*-*vpu* fusion gene did not change in HIV-1 infected individuals over time.

**Conclusions:**

Expression of a *rev1-vpu* fusion gene does not affect regular Rev and Vpu functions or alter HIV-1 replication in primary target cells. Since there is no evidence for increased replication fitness *of rev1-vpu* encoding viruses, this polymorphism likely emerged in the context of other mutations within and/or outside the *rev1-vpu* intergenic region, and may have a neutral phenotype.

## Introduction

HIV-1 protein synthesis is a tightly regulated process that involves the generation of more than 100 viral mRNA species [1]. These transcripts are translated into the structural proteins Gag, Pol and Env as well as two regulatory (Tat, Rev) and four accessory proteins (Vif, Vpr, Vpu, Nef). In addition to these nine proteins, several studies have reported the existence of fusion proteins, albeit only in tissue culture-propagated strains of HIV-1 [1–6]. These fusion proteins are the result of alternative splicing, when exons of regular and/or alternative open reading frames (ORFs) are brought together [1–6]. For example, the first exon of *tat* (*tat1*) and the second exon of *rev* (*rev2*) can be joined via cryptic exons in *env*. Depending on the *env* exon used, splicing results in the synthesis of a 26 kDa protein designated TNV or a 28 kDa fusion called TEV [2,3]. Similar to the parental Tat, these chimeric proteins are able to activate LTR-dependent transcription [2,3]. However, mutational analyses of the respective splice acceptor and donor sites have shown that TNV expression is not essential for HIV-1 replication *in vitro* [5]. Most recently, a previously unappreciated class of 1 kb transcripts was identified [1], some of which encoded novel viral proteins, including an unstable fusion protein comprising parts of Rev and Nef (Ref) as well as a Tat variant that contained 25 additional amino acids fused to its C-terminus (Tat^8c) [1]. In addition, fusion proteins comprising parts of Tat, Rev, and Vpu (Vpt) as well as Tat and gp41 (p17^tev^) have been described [4,6]. Whereas Tat^8c and p17^tev^ exert some Tat activity, the functions of Vpt and Ref remain unknown [1,4,6].

**Fig 1:**
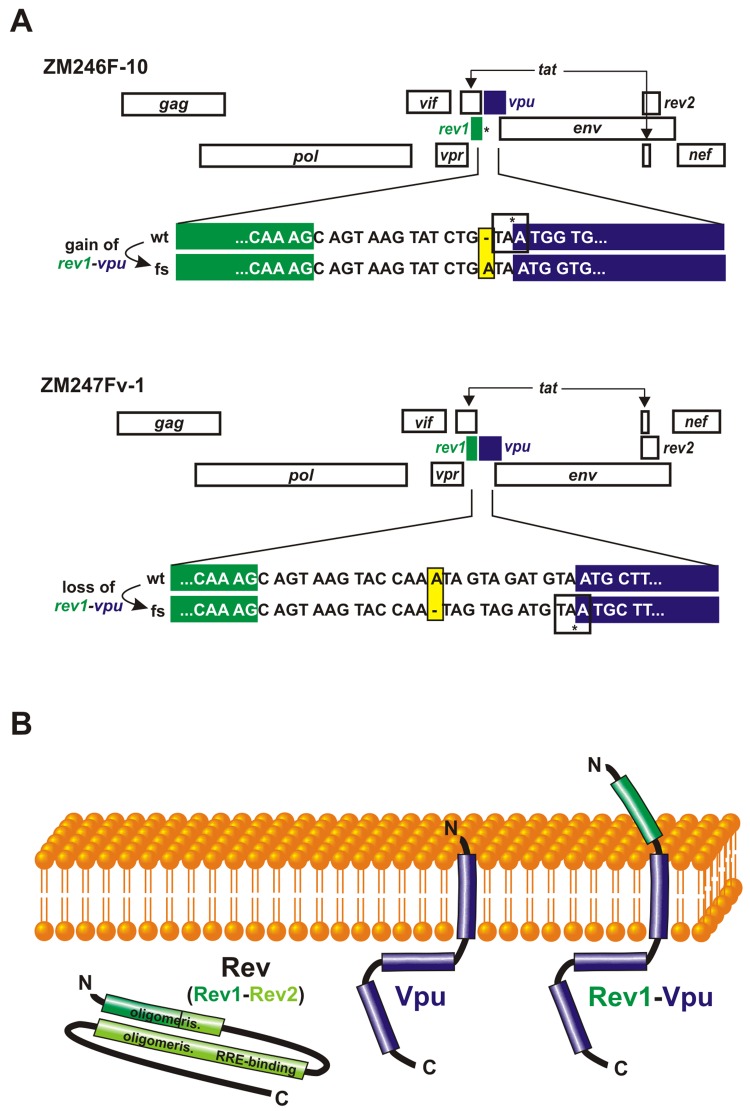
Varying genomic organization of HIV-1 in the intergenic region between *rev1* and *vpu*. (A) The relative position of genes within HIV-1 subtype C clones ZM246F-10 (upper panel) and ZM247Fv-1 (lower panel) is shown. Whereas ZM246 *rev1* (green) and *vpu* (blue) lie within different reading frames and are separated by an intervening stop codon (*), ZM247 encodes a *rev1-vpu* fusion gene. Frameshift mutations that were introduced to generate or disrupt *rev1-vpu* in ZM246 and ZM247, respectively, are highlighted in yellow. (B) Putative topology of the Rev1-Vpu fusion protein and its parental proteins Rev (green) and Vpu (blue).

Alternative splicing is not the only mechanism that can generate unusual fusion proteins in HIV-1. In 2010, we reported an HIV-1 gene arrangement in which *rev1* and *vpu* genes were present in the same reading frame without an intervening stop codon (Fig 1A) [7]. Analysis of the deduced protein sequence of this gene fusion suggests that it spans the plasma membrane like Vpu, but may contain an additional extracellular Rev-derived N-terminal domain (Fig 1B). Canonical Vpu promotes efficient release of infectious virions by decreasing the cell surface levels of CD4 [8] and counteracts the host restriction factor tetherin [9,10]. Furthermore, Vpu down-modulates the NK and NKT cell activating receptors NTB-A and CD1d [11,12] and blocks antiviral gene expression by inhibiting the activation of NF-кB [13–16]. Since Rev1-Vpu contains the entire Vpu protein sequence, the fusion protein could exert some of these functions, but may also have a negative effect. In addition, Rev1-Vpu may affect Rev activity, although it lacks the C-terminal part of Rev (Fig 1B). Canonical Rev bypasses the normal checkpoint of RNA splicing by mediating the nuclear export of incompletely spliced viral mRNAs. It performs this function by binding to the Rev responsive element (RRE) present in unspliced mRNAs [17]. Both the nuclear localization signal and the hydrophobic activation domain of Rev, which are required for RRE binding, fall within the second exon of Rev (*rev2*) that is absent from Rev1-Vpu. Thus, it is highly unlikely that Rev1-Vpu is able to perform this function. However, the fusion protein contains parts of the Rev oligomerization domain and may interfere with regular Rev activity. Here, we investigated whether the presence of a *rev1*-*vpu* fusion gene influences known Rev or Vpu functions and whether expression of this fusion protein alters the replicative capacity of HIV-1 in physiologically relevant target cells.

## Results

### Fusion gene containing HIV-1 proviruses express a Rev1-Vpu fusion protein

**Fig 2:**
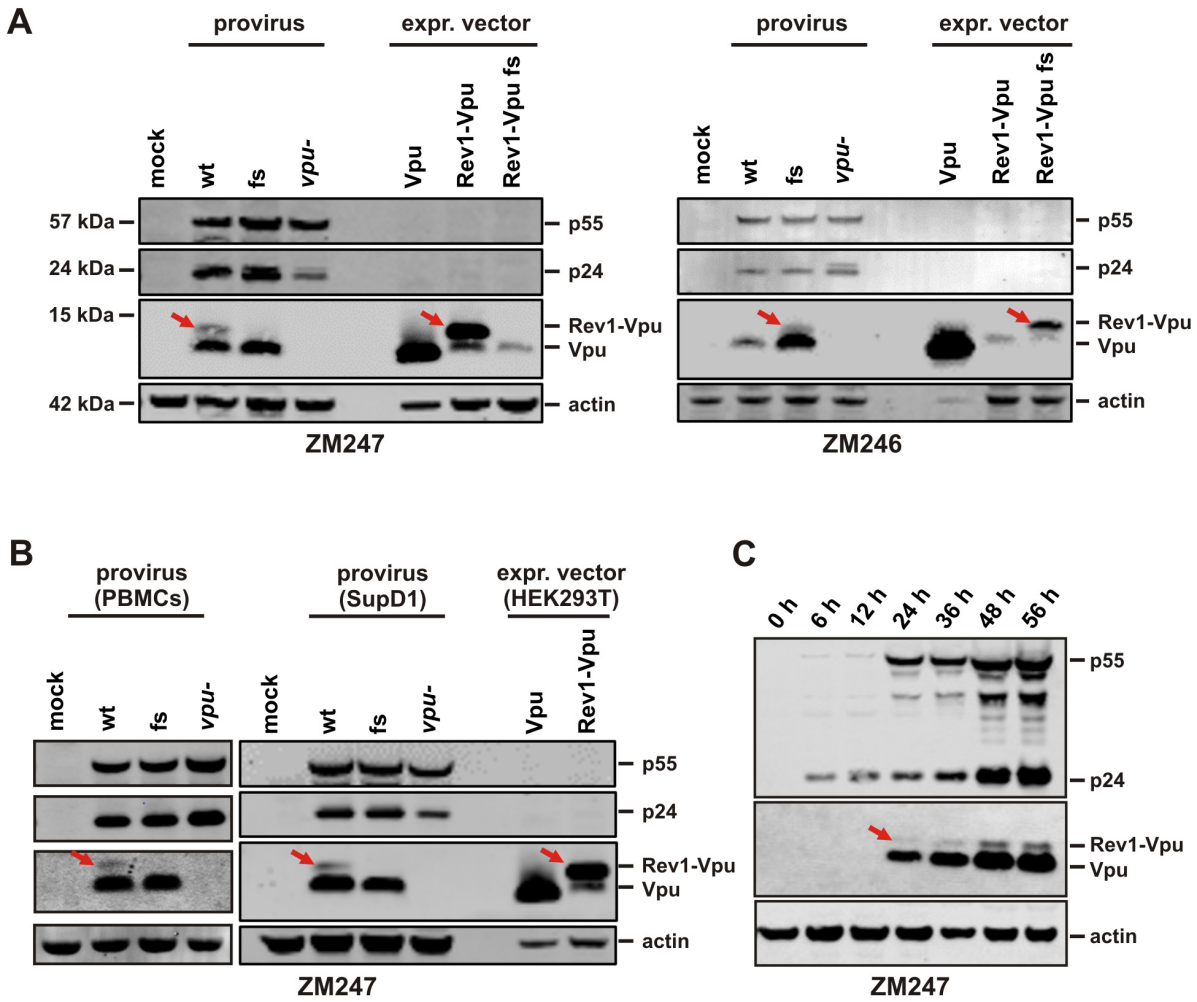
Expression of the Rev1-Vpu fusion protein. (A) Western blot analysis of HEK293T cells co-transfected with the proviral clones described in Fig 1A or a *vpu*-deficient mutant thereof. Expression vectors containing *rev1*-*vpu* or *vpu* cassettes served as size controls. Vpu and Rev1-Vpu were detected with an antiserum raised against ZM247 Vpu. (B, C) Expression of Vpu and Rev1-Vpu in ZM247-infected PBMCs or SupD1 cells. Bands representing the Rev1-Vpu fusion protein are highlighted by red arrows. Detection of p55, p24 and actin served as internal controls.

To investigate the expression pattern and function of Rev1-Vpu, we took advantage of two subtype C infectious molecular clones (ZM246F-10 and ZM247Fv-1, from now on referred to as ZM246 and ZM247) that represent transmitted founder viruses [18,19]. Whereas *rev1* is separated from *vpu* by a stop codon in the ZM246 clone, ZM247 encodes a *rev1*-*vpu* fusion gene (Fig 1A). To examine the effect of the *rev1-vpu* polymorphism on virus replication, we introduced frameshift (fs) mutations in the *rev1-vpu* intergenic regions of both ZM246 and ZM247, resulting in the gain and loss of the fusion gene, respectively (Fig 1A). Western blot analyses showed that HEK293T cells transfected with these proviral constructs expressed high levels of regular Vpu irrespective of the presence or absence of the fusion gene (Fig 2A). For the two viruses that encoded a *rev1-vpu* fusion gene (ZM247 wt and ZM246 fs), an additional Vpu-antiserum reactive protein of ~14 kDa molecular weight was observed (Fig 2A). Since this protein was absent from Western blots of the clones (ZM246 wt and ZM247 fs) in which *rev1* and *vpu* were separated by an intervening stop codon, it is highly likely that the 14 kDa band represents the Rev1-Vpu fusion protein. This conclusion was confirmed by the fact that its molecular weight was identical to that of Rev1-Vpu translated from expression vectors (Fig 2A).

To determine whether the Rev1-Vpu fusion protein was also expressed in CD4+ target cells, we infected peripheral blood mononuclear cells (PBMCs) and the T cell line SupD1 [16] with ZM247 wild type and frameshift viruses. Similar to the results obtained in transfected HEK293T cells, the wild type virus expressed the Rev1-Vpu fusion protein, but at much lower levels compared to regular Vpu (Fig 2B). Since the generation of *vpu*/*env* and *rev* transcripts is temporally regulated [1] and other fusion proteins, such as TEV, appear to be expressed prior to the production of their parental proteins [3], we examined the expression kinetics of Rev1-Vpu and Vpu in infected SupD1 cells. Western blot analyses of infected cell lysates from seven different time points identified the same ratio of Vpu and Rev1-Vpu over time (Fig 2C). These results show that *rev1-vpu* containing proviruses express the fusion protein, but at a much lower level compared to canonical Vpu.

### Co-expression of Rev1-Vpu and Vpu results in less efficient down-modulation of CD4, tetherin, CD1d and NTB-A in transfected cells

**Fig 3:**
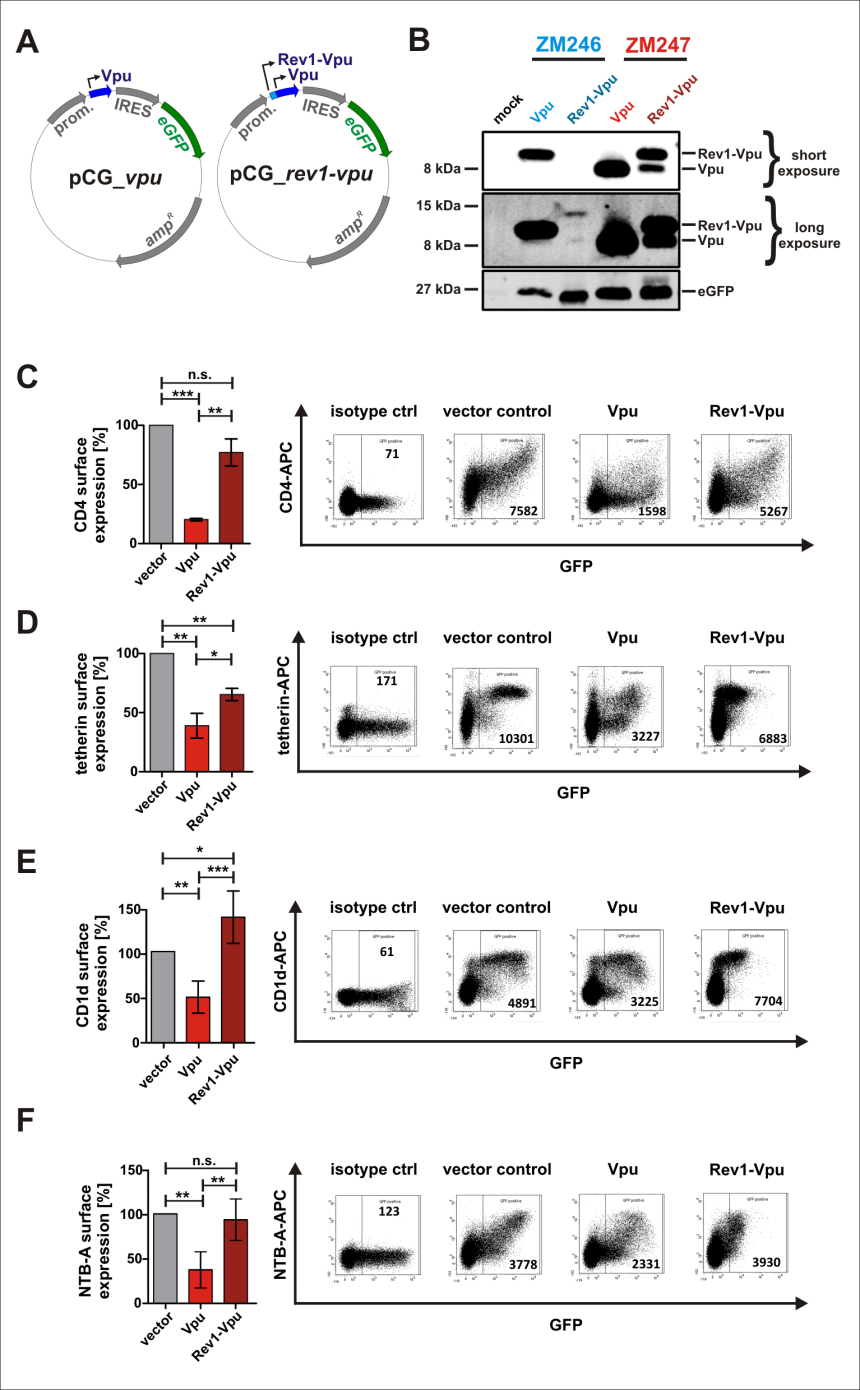
Functional activity of Rev1-Vpu expressed from pCG expression plasmids. (A) CMV-IE promoter-based pCG expression vectors containing *vpu* (left panel) or the *rev1-vpu* fusion gene (right panel). An enhanced version of the green fluorescent protein (eGFP) is co-expressed via an IRES. (B) Expression of Rev1-Vpu and Vpu in HEK293T cells transfected with the indicated pCG vectors. A Vpu-specific antiserum was used for detection. eGFP was detected to check transfection efficiencies. (C-F) FACS analysis of (C) CD4, (D) tetherin, (E) CD1d or (F) NTB-A receptor modulation by ZM247 Vpu and Rev1-Vpu. HEK293T cells were transfected with expression vectors for the respective surface receptor and Vpu or Rev1-Vpu. 40 h post transfection, surface receptor levels were monitored by two-color flow cytometry. Dot plots indicating the gating strategy are shown in the right panels. Bar diagrams summarizing three to five independent experiments +/- SD are shown on the left (***p<0.001; **p<0.01; *p<0.05; n.s. not significant).

To investigate whether Rev1-Vpu exerts any of the functions ascribed to its parental Vpu protein, we cloned the ORFs of *rev1-vpu* and *vpu* of ZM246 fs and ZM247 wt into the CMV promoter-based pCG expression vector [20] (Fig 3A). As expected, ZM246 and ZM247 Vpu proteins were efficiently expressed in transfected HEK293T cells (Fig 3B). Due to leaky scanning, the vector containing the ZM247 fusion gene did not only express Rev1-Vpu but also regular Vpu at a similar level (Fig 3B). Surprisingly, only trace amounts of Rev1-Vpu were detected in cells transfected with the vector containing the fusion gene of ZM246 fs (Fig 3B). Since this low expression levels precluded any meaningful analyses of ZM246 Rev1-Vpu, only the ZM247 expression vectors were used for further experiments.

Flow cytometric analyses of transfected HEK293T cells revealed that ZM247 Vpu alone reduced the cell surface levels of CD4, tetherin, CD1d and NTB-A more efficiently than the combination of Vpu and Rev1-Vpu (Fig 3C, 3D, 3E and 3F). Thus, in the context of these transfection experiments the fusion protein either lacked these Vpu functions and/or exerted a dominant negative effect.

### Proviruses expressing a Rev1-Vpu fusion protein do not exhibit a defect in Vpu function

**Fig 4:**
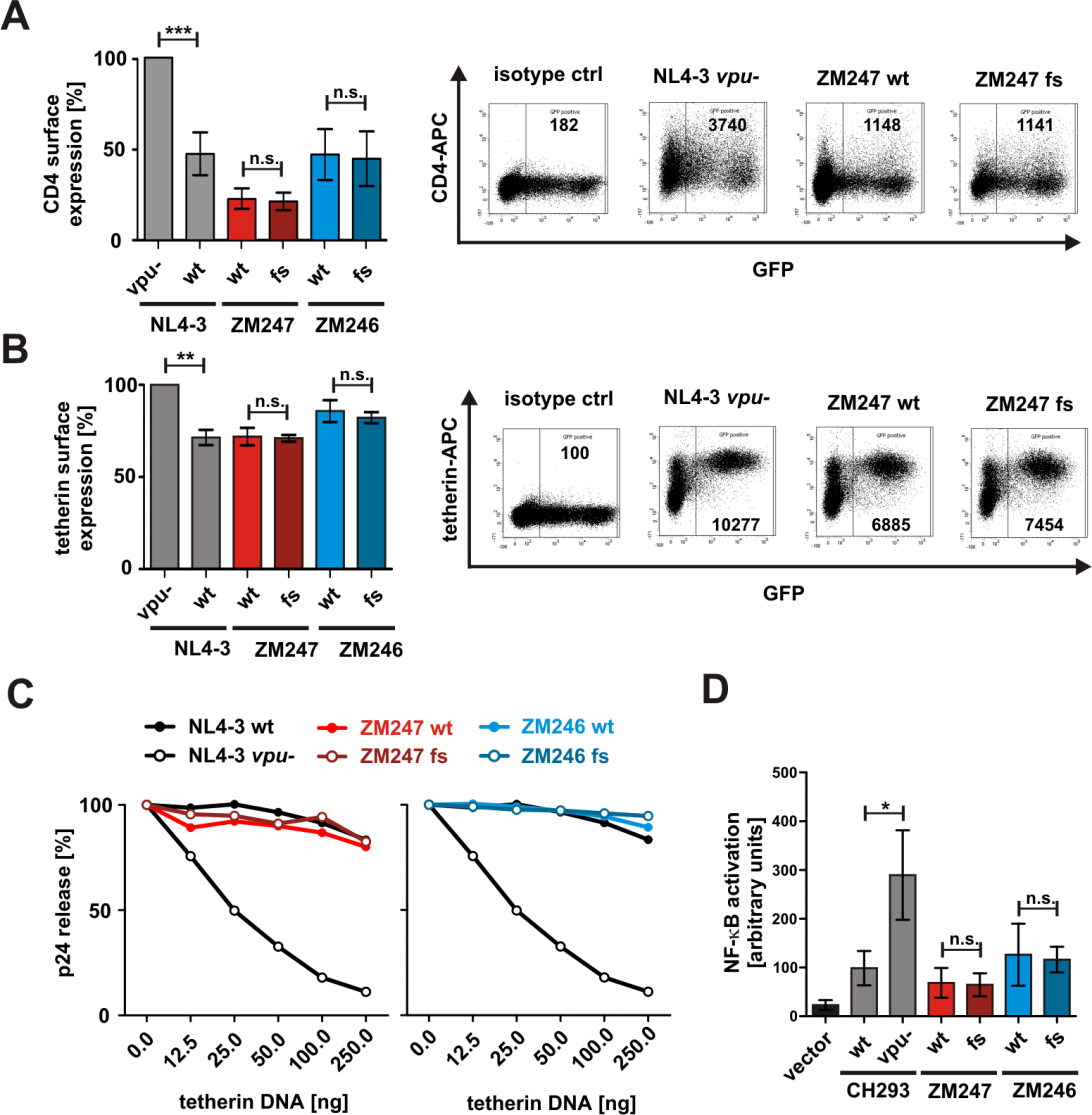
Vpu function in isogenic viruses differing only in their ability to express Rev1-Vpu. (A, B) FACS analysis of surface expression levels of (A) CD4 and (B) tetherin on HEK293T cells co-transfected with the indicated proviral constructs and expression vectors for the respective surface molecule. Dot plots indicating the gating strategy are shown in the right panels. Bar diagrams summarizing three to six independent experiments +/- SD are shown on the left. (C) p24 release from HEK293T cells co-transfected with the indicated proviral constructs and increasing amounts of a tetherin expression plasmid. 40 h post transfection, the amounts of cell-associated and cell-free p24 were analyzed by ELISA. Relative release was calculated as ratio of p24 in the supernatant to total p24. The means of at least three independent experiments are shown. (D) Activation of NF-кB by viruses harboring the fusion polymorphism or not. HEK293T cells were co-transfected with the indicated proviral clones, and an NF-кB-responsive firefly luciferase reporter construct. 40 h post transfection, firefly luciferase activity was determined and normalized to the activity of a *Gaussia* luciferase control plasmid. The means of three independent experiments +/- SD are shown (***p<0.001; **p<0.01; *p<0.05; n.s. not significant).

Since the transfection studies resulted in the overexpression of both Vpu and Rev1-Vpu, we next analyzed the fusion protein in the context of proviral clones. To test whether the presence of the fusion gene affected known Vpu functions, we co-transfected HEK293T cells with both wt and fs versions of the infectious molecular clones of ZM246 and ZM247, together with expression vectors for human tetherin or CD4. The lab-adapted HIV-1 NL4-3 clone and a *vpu*-deficient derivative served as controls. Two days post transfection, surface expression of CD4 and tetherin were analyzed by flow cytometry. As expected, NL4-3, ZM246 and ZM247 wild type viruses reduced the surface levels of CD4 and tetherin more efficiently than the NL4-3 control lacking *vpu* (Fig 4A and 4B). However, the presence or absence of a *rev1-vpu* fusion gene had no effect on the efficiency of CD4 and tetherin down-modulation by the proviral ZM246 and ZM247 constructs (Fig 4A and 4B). Since tetherin inhibits the egress of newly formed virions, we also quantified p24 release in the presence of increasing amounts of this restriction factor. Consistent with the results obtained by flow cytometry, the fusion gene had no appreciable effects on the efficiency of virus release (Fig 4C).

Vpu also blocks antiviral gene expression by inhibiting NF-кB signaling [13–16]. Using a dual luciferase reporter assay [16], we compared the activation of this transcription factor by viruses that encoded the *rev1-vpu* fusion versus those that did not. As expected, a *vpu*-deficient variant of HIV-1 CH293 induced significantly higher levels of activated NF-кB than the respective wild type control (Fig 4D). In contrast, suppression of NF-кB activation did not differ between the wild type and frameshift variants of ZM246 and ZM247 (Fig 4D). In summary, the presence of a *rev1-vpu* fusion gene in the HIV-1 provirus had no effect on known Vpu functions.

### The *rev1-vpu* polymorphism does not affect Rev-dependent gene expression

**Fig 5:**
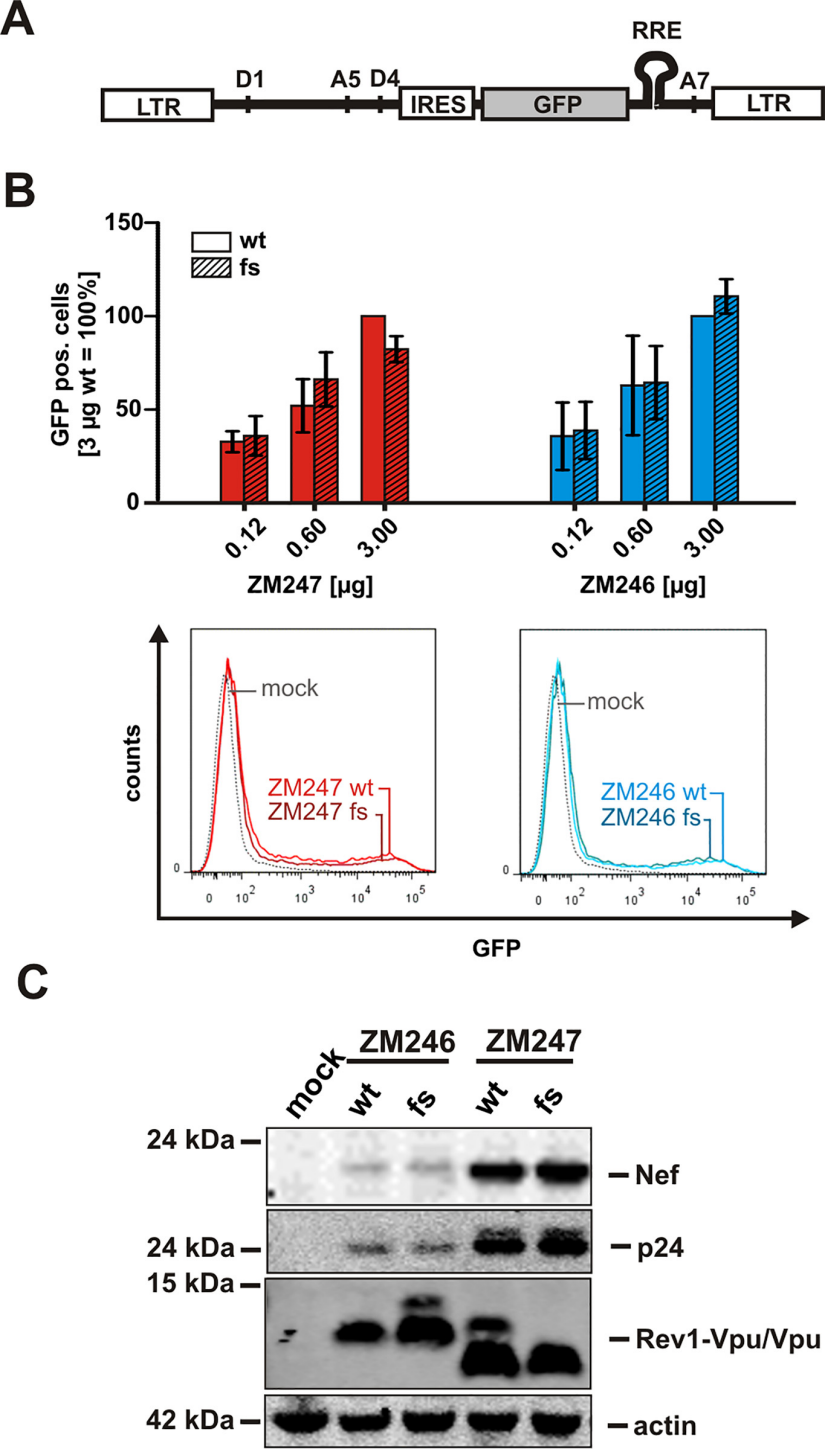
Rev function in isogenic viruses differing only in their ability to express Rev1-Vpu. (A) Gene arrangement of a reporter construct expressing GFP in a Rev-dependent manner. The *GFP* ORF is flanked by splice donor site 4 (D4) and splice acceptor site 7 (A7). Rev mediates the export of intron-containing GFP expressing mRNA via binding to the RRE. (B) Rev-dependent gene expression was determined by co-transfection of HEK293T cells with increasing amounts of the indicated molecular clones of HIV-1, the GFP reporter construct and a BFP expressing control plasmid. 40 h post transfection, GFP expression levels of BFP positive cells were analyzed by flow cytometry. Examples of primary FACS data are shown in the lower panel. (C) Western blot, showing Rev-dependent expression of p24-capsid and Rev-independent expression of Nef in HEK293T cells transfected with the indicated proviral constructs.

Since the RRE binding domain of the Rev protein is encoded by its second exon (*rev2*), we did not expect Rev1-Vpu to mediate the nuclear export of incompletely spliced mRNAs. Nonetheless, Rev1 comprises parts of the Rev oligomerization domain (Fig 1B), raising the possibility that Rev1-Vpu may exert some dominant negative activity on Rev function. To test this, we took advantage of a previously described reporter construct that expresses GFP in a Rev-dependent manner [21]. This construct contains an IRES GFP cassette that is flanked by splice donor and acceptor sites (Fig 5A). Thus, GFP is only expressed in the presence of Rev, which binds to the RRE within the IRES GFP cassette. To analyze whether Rev1-Vpu has an effect on Rev-mediated RNA export, we co-transfected HEK293T cells with the Rev reporter construct as well as the wild type or frameshift variants of ZM246 and ZM247. As expected, GFP reporter gene expression was induced by increasing amounts of HIV-1 (Fig 5B). However, we did not observe significant differences in Rev activity between wild type and frameshift-containing viruses, indicating that the fusion protein, which was expressed in both ZM246 fs and ZM247 wt infected cells (Fig 5C), does not alter Rev function (Fig 5B). Consistent with this, Rev-dependent Gag expression was proportional to the expression of the early protein Nef, which is translated in a Rev-independent manner (Fig 5C).

### The *rev1-vpu* polymorphism does not enhance HIV replication fitness

**Fig 6:**
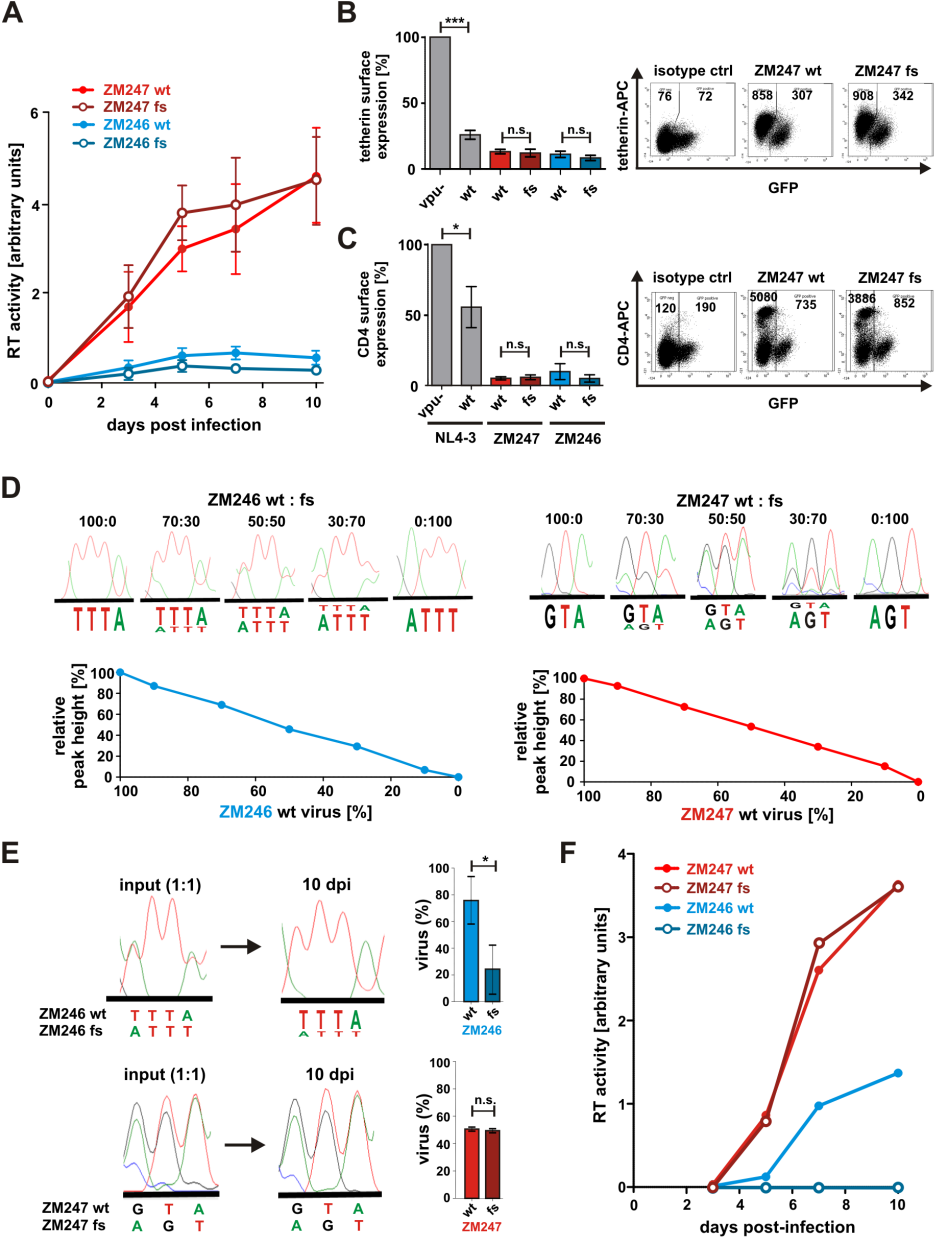
Effect of Rev1-Vpu expression on HIV-1 replication in PBMCs and tonsillar explant cultures. (A) PHA-stimulated PBMCs were infected with adjusted amounts of the indicated viruses. Virus replication was monitored by analyzing RT-activity in the supernatant. The means of three independent experiments +/- SEM are shown. (B, C) Surface expression levels of (B) tetherin and (C) CD4 were determined by flow cytometry at day 3 post infection. Infected cells were identified by intracellular p24 staining after surface staining of CD4 or tetherin. Dot plots indicating the gating strategy are shown in the right panels. Bar diagrams summarizing four to five independent experiments +/- SD are shown on the left. (D) Sequence analyses of viral mixtures. Wt and fs mutant virus stocks were normalized for infectivity, mixed at the indicated ratios, and the *rev1-vpu* region was sequenced after bulk amplification of cDNA. The lower panels show respective standard curves. The peak fluorescence of the T residue at position 1 (ZM246 wt) and the A residue at position 3 (ZM247 wt) is expressed as a fraction of the total fluorescence (relative peak height). (E) Sequence chromatograms of 1:1 input mixtures and viral cultures 10 days post infection (dpi). Percentages of wt and fs sequences displayed in the right panels were calculated from the standard curves shown in (D). (F) Human tonsil explant cultures were infected and analyzed as described in (A). One representative experiment for one of three independent donors is shown (***p<0.001; **p<0.01; *p<0.05; n.s. not significant).

Although the *rev1-vpu* polymorphism did not affect known Rev or Vpu functions, we considered the possibility that it may affect viral replication by other mechanisms. To address this, PHA-stimulated PBMCs were infected with equal tissue culture infectious doses (TCIDs) of ZM247 and ZM246 strains that differed in their ability to express Rev1-Vpu. Although ZM247 replicated to higher titers than ZM246 [19], quantification of reverse transcriptase activity in the cell culture supernatants revealed only minor differences between the wild type and frameshift-containing viruses (Fig 6A). For ZM247, the frameshift mutant lacking *rev1-vpu* replicated slightly more efficiently, while the opposite was true for ZM246 (Fig 6A), although these differences were not statistically significant. At day 3 post-infection, surface expression levels of tetherin and CD4 were quantified by flow cytometry. In agreement with the data obtained in transfected HEK293T cells, the efficiency of CD4 and tetherin surface down-modulation in HIV-1 infected primary cells was not affected by the presence of a fusion gene (Fig 6B and 6C).

To not miss subtle differences in viral replication, we performed a direct competition assay where PBMCs were simultaneously infected with equal TCIDs of both wild type and frameshift viruses. Ten days post infection, viral RNA was isolated from the cell culture supernatants, reverse transcribed, and the *rev1-vpu* region was sequenced in bulk. The relative fluorescence of the wild type and frameshift genomes in the sequence chromatograms reflected their input ratios and thus allowed the generation of standard curves (Fig 6D). The analysis of virus mixtures before and after culture in PBMCs showed that the ZM246 wild type virus outpaced its frameshifted counterpart. At the end of the competition assay, the majority (74% +/- 18%) of the isolated ZM246 genomes lacked the introduced fusion mutation (Fig 6E). In contrast, ZM247 wild type, which naturally encodes the fusion gene, and its corresponding frameshift mutant replicated with similar efficiencies (Fig 6E).

To examine possible effects of the *rev1-vpu* fusion gene under conditions that more closely resemble HIV-1 infection *in vivo*, we also infected human tonsillar explant cultures that promote HIV-1 replication in the absence of exogenous stimuli [22]. In agreement with the results obtained in PBMCs, ZM247 wild type and frameshift constructs replicated with similar kinetics (Fig 6F). Interestingly, however, the newly introduced *rev1-vpu* polymorphism abrogated the ability of ZM246 to replicate in these cultures (Fig 6F).

### Mutations in the *rev1-vpu* intergenic region affect *env* expression

**Fig 7:**
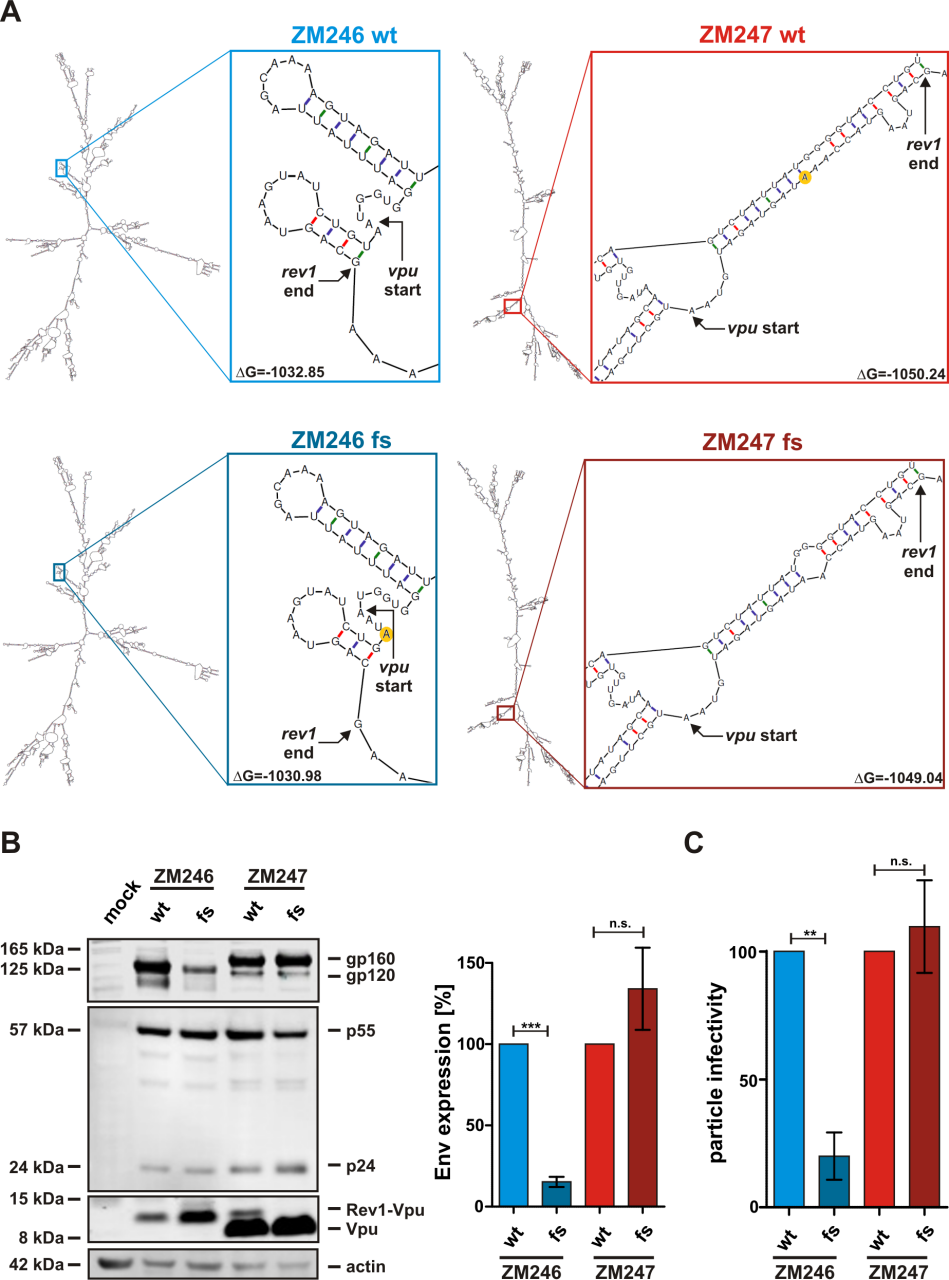
Effect of *rev1-vpu* frameshift mutations on mRNA structure and Env expression. (A) mRNA structures of ZM246 wt, ZM246 fs, ZM247 wt and ZM247 fs were predicted using the Mfold web server for nucleic acid folding and hybridization prediction [37]. The putative mRNA structures of full length *rev/vpu/env* enconding mRNAs (using splice sites D1 and A4c) are shown on the left of each panel. Close-ups of the *rev1-vpu* intergenic region and the minimum free energy ΔG are shown on the right. The nucleotides that are deleted in ZM247 wt and inserted in ZM246 fs are highlighted in yellow. (B) Western blot analysis of HEK293T cells co-transfected with the proviral clones described in Fig 1A. gp160 and gp120 were detected using an antiserum raised against HIV-1 M subtype C 96ZM651. A representative Western blot is shown on the left. Total Env expression (gp120+gp160) was normalized to total Gag expression (p55+p24). The mean values (+/- SEM) of four independent transfections are shown on the right. (C) Particle infectivity was determined by infecting TZM-bl reporter cells with equal amounts of p24. Three days post infection, β-galactosidase activity was measured. The mean values (+/- SEM) of triplicate infections from two to four independent transfections are shown (***p<0.001; **p<0.01; n.s. not significant).

Since the frameshift mutation significantly reduced the replicative capacity of ZM246 without affecting its Vpu or Rev activities, we hypothesized that the presence of a fusion gene may be just an epiphenomenon of other adaptive mutations. Notably, the *rev1-vpu* intergenic region contains minimal ORFs as well as potential shunt or IRES elements that regulate the expression of Vpu and Env [23,24]. Anderson and colleagues suggested that specific RNA structures upstream of *env* may enable discontinuous ribosome scanning and regulate Env expression from multicistronic mRNA [24]. Indeed, *in silico* structural analyses predicted several interior and multi-branched loops as well as hairpin structures in the *rev1-vpu* intergenic regions of ZM246 and ZM247 (Fig 7A, top panels). The frameshift mutations generating or disrupting the *rev1-vpu* fusion gene, respectively, did not break up these structures and had only minor effects on the folding of *rev/vpu/env* encoding mRNA (Fig 7A, bottom panels). Nevertheless, Env expression levels of ZM246 fs were reduced by about 85% compared to its parental control (Fig 7B). In contrast to that, Env glycoprotein levels of ZM247 remained unaffected by the frameshift mutation (Fig 7B). In agreement with cellular Env protein levels, particle infectivity of ZM246 fs was also decreased by about 80% whereas particle infectivity of ZM247 was not affected by the frameshift (Fig 7C).

### No evidence of acquisition or loss of the *rev1-vpu* polymorphism in infected individuals over time

To estimate the percentage of HIV-1 strains harboring the *rev1-vpu* polymorphism we analyzed 2622 subtype A, C and CRF sequences from the Los Alamos HIV Sequence Database (http://www.hiv.lanl.gov/). Screening these sequences, each of which represented a different HIV-1 strain, for the fusion gene, we found 3% of clade A (11/396), 16% of clade C viruses (136/864), and 3% of A/C recombinants (42/1362) to harbor *rev1* and *vpu* in the same uninterrupted reading frame. Since these clades have been estimated to account for as many as 75% of all HIV-1 infections worldwide [25], it seems clear that a significant fraction of naturally occurring HIV-1 strains encode a *rev1-vpu* fusion gene. To examine whether the *rev1-vpu* fusion gene was under positive or negative selection during the course of HIV-1 infection, we analyzed the viral quasispecies in eight longitudinally sampled patients who were acutely infected with clade A or C strains [26,27]. Previous studies had shown that all of these patients were infected with single transmitted founder viruses, two of which encoded the *rev1-vpu* fusion gene, while six others did not (Table 1). Interestingly, the presence or absence of this polymorphism remained stable over time and we did not observe any obvious association with median CD4 counts or virus setpoint. Analysis of the evolving quasispecies in each patient failed to identify any example of viral mixtures, despite the generation of up to 101 single template derived sequences from multiple time points spanning as many as 96 weeks of observation (Table 1). Thus, there was no evidence for the acquisition or loss of the *rev1-vpu* fusion gene in subtype A and C infected individuals over time.

**Table 1:** Frequency of the *rev1-vpu* polymorphism in HIV-1 quasispecies of infected individuals over time.

patient	subt.	number of TF variants	time points sequenced	total weeks studied	SGS^a^ week 0	SGS^a^ week 2/3	SGS^a^ week 8	SGS^a^ week 24	SGS^a^ week 36	SGS^a^ week 48	SGS^a^ week 60	SGS^a^ week 96	fraction SGS with *rev1-vpu* fusion	accession numbers	ref.
R880F	A	1	4	49	-	-	10	3	11	11	-	-	**0/35**	KP223797-809, KP223815-26, KP223834-43	[26]
R463F	A	1	5	48	-	14	8	8	8	9	-	-	**0/47**	KP223729-75	[26]
705010185	C	1	5	60	27	11	27	15	-	-	16	-	**0/96**	JX973075-170	[27]
706010164	C	1	5	60	37	10	23	11	-	-	20	-	**101/101**	JX973234-334	[27]
705010162	C	1	5	60	14	17	7	9	-	-	23	-	**70/70**	JX972986-98, JX973019-74, JX974246	[27]
704010042	C	1	6	96	9	18	6	12	-	-	24	25	**0/94**	JX972838-930, JX974245	[27]
705010198	C	1	4	60	10	10	28	-	-	-	15	-	**0/63**	JX973171-233	[27]
703010256	C	1	6	96	10	12	27	10	-	-	15	24	**0/98**	JX972739-72, JX972774-837	[27]

^a^ single genome sequences (SGS) per sample visit

## Discussion

**Fig 8:**
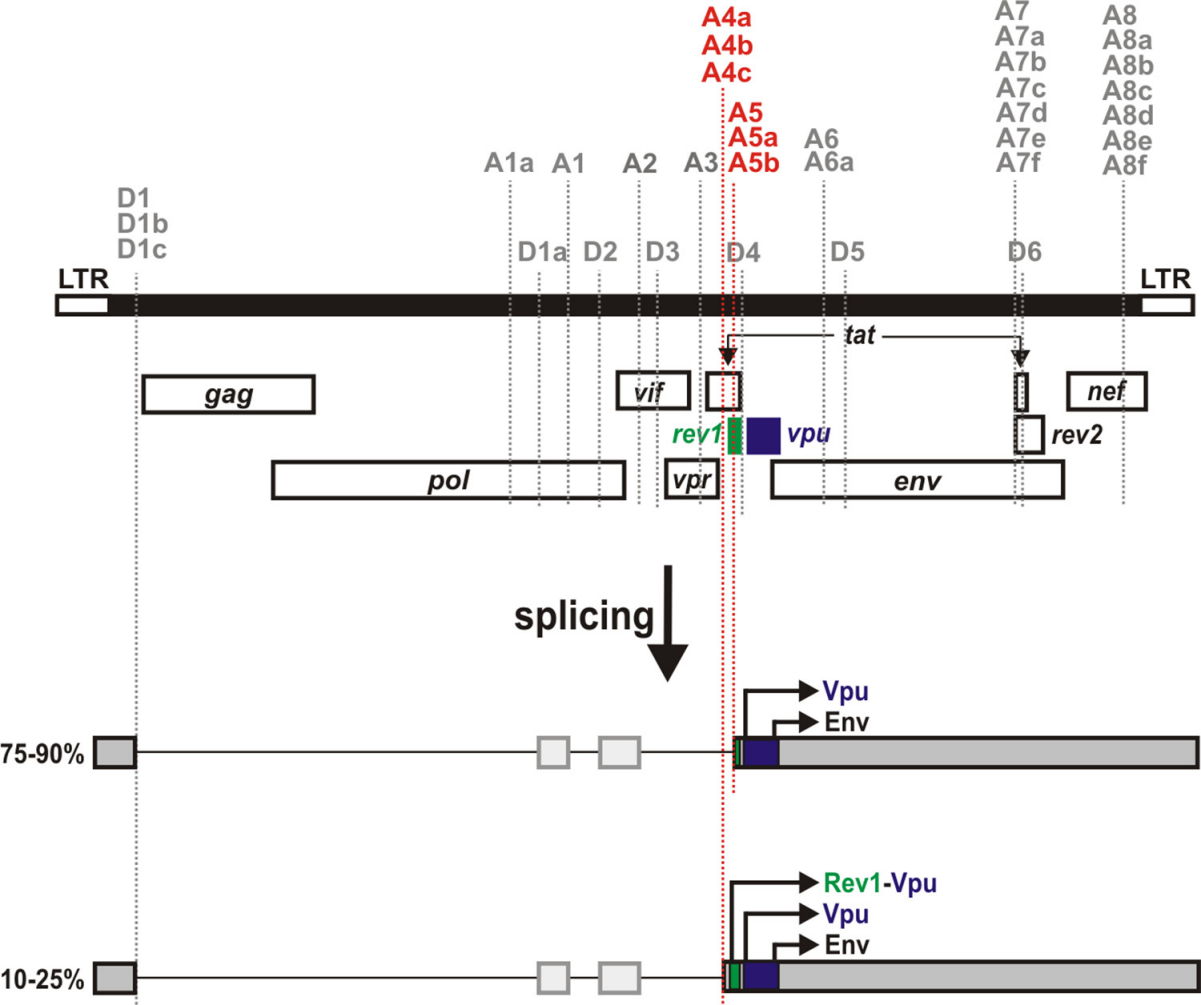
Splice sites generating Rev1-Vpu encoding mRNA. The HIV-1 genome (black) and nine ORFs encoding structural, regulatory and accessory proteins are depicted on top. Splice donor (D1-6) and acceptor (A1-8) sites are indicated by dotted lines. mRNAs encoding Rev1-Vpu, Vpu and Env are shown in grey. Depending on the cell type and time point post infection, 75–90% of Vpu and Env encoding mRNA species fail to express Rev1-Vpu since the usage of splice acceptor site A5 removes an intron containing the initiation codon of *rev1*. Only 10–25% of the Vpu encoding mRNAs have the potential to be translated into Rev1-Vpu since splice acceptor sites A4a, A4b and A4c retain the complete first exon of *rev*.

All previously described HIV-1 fusion proteins, including TEV, TNV, Vpt, p17^tev^, Ref and Tat^8c were discovered in tissue culture-propagated HIV-1, when alternative splicing was identified to generate mRNAs that contained exons from different canonical and/or non-canonical open reading frames [1–4]. To our knowledge, *rev1-vpu* is the only primate lentiviral fusion gene that is present in the genome of naturally occurring viral strains [7], thus warranting investigation of its potential functions. Here, we show that a Rev1-Vpu fusion protein is expressed in primary target cells, albeit at much lower levels than the cognate Vpu protein. This is due to the fact that a major splice acceptor (A5) generates mRNAs that lack the initiation codon of *rev1* [28,29], which prevents the expression of the fusion protein (Fig 8). Only about 10% to 25% of *vpu* and *env* encoding mRNAs use upstream splice acceptors (A4a, b or c) that retain the complete first exon of *rev* and thus have the potential to express the fusion protein [1,28] (Fig 8). However, even these (*rev1*-*vpu* encoding) mRNAs may express regular Vpu, since the *rev1* initiation codon may be skipped due to a weak Kozak sequence (leaky scanning) [23,29–31] or by-passed due to the RNA secondary structure in this region (ribosomal shunting) [23,31]. The very low Rev1-Vpu expression levels may explain why the function of the parental Vpu and Rev proteins remain unaltered: viruses differing only in their ability to express Rev1-Vpu down-modulated CD4, counteracted tetherin and inhibited the activation of NF-кB with similar efficiencies. Furthermore, the fusion protein did not exert a dominant negative activity on Rev-mediated mRNA export, although Rev1-Vpu contains parts of the Rev oligomerization domain.

Competition assays and replication kinetics in primary PBMCs as well as tonsillar explant cultures revealed that the loss of a naturally occurring fusion gene did not affect the replication capacity of the corresponding virus (ZM247). Interestingly, however, creation of a fusion gene in a virus that did not naturally encode one (ZM246) impaired its replication potential in these same cultures. The poor replication of the ZM246 frameshift mutant could be ascribed to decreased Env expression levels and thus reduced particle infectivity. Although mRNA folding was hardly affected by the frameshift mutation, the insertion of a nucleotide in the *rev1-vpu* intergenic region may have disrupted unknown shunting or IRES elements that regulate Env expression [24]. Alternatively, the mutation may affect linear ribosome scanning by increasing the Kozak strength of *vpu*. In agreement with the latter hypothesis, Vpu levels were slightly increased in the frameshift mutant of ZM246 compared to its wild type counterpart.

In summary, these findings raise the possibility that the emergence of a *rev1*-*vpu* fusion gene is merely a side effect of mutations that regulate Env glycoprotein expression. While higher Env levels enhance particle infectivity, lower levels may serve as an immune evasion mechanism to reduce antibody accessibility. Thus, primate lentiviruses have to strike a fine balance between optimizing virion infectivity and escaping the humoral immune response of their host. The generation of a *rev1-vpu* fusion gene might not be essential to optimize *env* expression but may be tolerated as an epiphenomenon since Rev1-Vpu is only expressed at very low levels and does not significantly affect known Rev or Vpu functions.

While our *in vitro* and *in vivo* data provide clear evidence against a general fitness advantage of *rev1*-*vpu* containing viruses, they do not explain the apparent persistence of this mutation in a subset of subtype A and C strains. Since tissue culture based findings have obvious limitations, it is possible that the *rev1*-*vpu* fusion gene evolved in a subset of infected individuals in response to particular host and/or environmental pressures. However, the absence of examples of either the gain or loss of the fusion gene in eight longitudinally studied patients would suggest that such events, if they occur, are exceedingly rare. It thus seems most likely that the *rev1-vpu* fusion gene emerged as an epiphenomenon of other adaptive mutations, such as the optimization of *env* expression. Most HIV strains, however, may have taken another evolutionary road and achieved the same goal without generating a *rev1-vpu* fusion gene.

## Conclusions

We show that naturally occurring HIV-1 strains, including transmitted founder viruses, which encode the first exon of *rev* and *vpu* in the same reading frame, have the capacity to express a Rev1-Vpu fusion protein, albeit at much lower levels than regular Vpu. Importantly, this polymorphism neither affects known Vpu or Rev functions, nor does it affect viral replication in human PBMCs and lymphoid explant cultures. It thus seems likely that the *rev1*-*vpu* fusion gene emerged in the context of other mutations within and/or outside the *rev1-vpu* intergenic region, and may have a neutral phenotype.

## Methods

### Expression vectors

*Rev1-vpu*, *vpu*, *tetherin*, and *CD1d* genes were inserted via XbaI/MluI into the CMV promoter‐based pCG expression vector [20]. An IRES eGFP, IRES BFP or IRES DsRed2 cassette was inserted via BamHI so that the gene of interest is expressed together with the fluorophore from a single bi-cistronic mRNA. The human *CD4* gene was cloned into pcDNA3.1(+) via HindIII/XbaI; transcript variant 2 of human *NTB-A* was cloned into pQCXIP [11]. The pHIT/G vector expressing the vesicular stomatitis glycoprotein has been described previously [32]. An NF-кB firefly luciferase reporter plasmid containing three NF-кB binding sites was kindly provided by Dr. Bernd Baumann. A minimal promoter *Gaussia* luciferase construct was purchased from Clontech (#631909) and used for normalization. It contains the TATA-like promoter (pTAL) region from the Herpes simplex virus thymidine kinase (HSV-TK) that is not responsive to NF-кB. The Tat/Rev reporter vector pNL-GFP-RRE(SA) was obtained through the NIH AIDS Reagent Program, Division of AIDS, NIAID, NIH from Dr. John Marsh and Dr. Yuntao Wu [21].

### Proviral constructs

Proviral clones of HIV-M NL4-3, ZM246F-10 and ZM247Fv1 have been described previously [18,33]. Using PCR-mediated overlap extension, frameshift mutations were introduced in the intergenic region between *rev1* and *vpu* to either generate (ZM246F-10) or disrupt (ZM247Fv1) the *rev1-vpu* fusion gene. The frameshift mutations are identical to those described by Kraus *et al*. [7]. To generate *vpu*-deficient mutants, stop codons were introduced after the *vpu* start codon (ZM247: codons 3 and 4; ZM246: codons 4, 7 and 10).

### Cell culture and transfections

HEK293T and TZM-bl cells were maintained in Dulbecco’s modified Eagle medium (DMEM) supplemented with 10% FCS plus 2 mM glutamine, streptomycin (120 mg/ml), penicillin (120 mg/ml) and transfected by the calcium phosphate method. 293T cells were first described by DuBridge *et al*. [34] and obtained from ATCC. TZM-bl cells were obtained through the NIH AIDS Reagent Program, Division of AIDS, NIAID, NIH from Dr. John C. Kappes, Dr. Xiaoyun Wu and Tranzyme Inc [35]. SupD1 cells were maintained in RPMI medium supplemented with 10% FCS plus 2 mM glutamine, streptomycin (120 mg/ml), penicillin (120 mg/ml) and Hygromycin B (200 μg/ml). This cell line is a NF-кB reporter cell line derived from SupT1 cells [16]. PBMCs from healthy human donors were isolated using lymphocyte separation medium (Biocoll separating solution; Biochrom), stimulated for three days with phytohemagglutinin (PHA) (1 μg/ml), and cultured in RPMI-1640 medium with 10% fetal calf serum (FCS) plus 2 mM glutamine, streptomycin (120 mg/ml), penicillin (120 mg/ml) and 10 ng/ml interleukin 2 (IL-2) prior to infection. Human tonsils removed during routine tonsillectomies and not required for clinical purposes were dissected into 2 to 3 mm^3^ blocks and cultured in RPMI supplemented with 10% FCS, 2 mM glutamine, streptomycin (120 mg/ml), penicillin (120 mg/ml), gentamycin (50 mg/ml), timentin (310 μg/ml), 1% sodium pyruvate, 1% fungizone and 1% non-essential amino acids. Tissue blocks were cultured on collagen sponge supports at a liquid/air interface as previously described [22]. The use of human tonsillar tissue was approved by the Ethics Committee of the Ulm University Medical Center. All donors provided written consent.

### Western blotting

To monitor expression of Rev1-Vpu and other viral proteins, HEK293T cells, PBMCs, and SupD1 cells were lysed in M-PER buffer (Thermo Scientific) two days post transfection and infection, respectively. Cell lysates were separated in 4–12% Bis-Tris gels (Invitrogen) and transferred to PVDF membranes. Blots were probed with antibodies against Vpu, Nef or p24. The monoclonal anti-p24 antibody was purchased from abcam (Cat# 9071, dilution 1:2,000). The Vpu antiserum (Cat# 11942, dilution 1:5,000) was obtained through the NIH AIDS Reagent Program, Division of AIDS, NIAID, NIH from Drs. Beatrice H. Hahn and Matthias H. Kraus [7]. The Nef antiserum (Cat# 2949, dilution 1:200) was obtained through the NIH AIDS Reagent Program, Division of AIDS, NIAID, NIH from Dr. Ronald Swanstrom [36]. The Env antiserum was purchased from ABLinc (Cat# 5411, dilution 1:1000). For internal controls, blots were incubated with antibodies specific for GFP (polyclonal, abcam, Cat# 290, dilution 1:10,000) and β-actin (polyclonal, abcam, Cat# 8227, dilution 1:2,000). Subsequently, membranes were incubated with anti-mouse or anti-rabbit IRDye Odyssey antibodies and proteins were detected using a LI-COR Odyssey scanner.

### Flow cytometry

To determine the effect of the *rev1-vpu* fusion gene on cell surface protein expression, HEK293T cells were transfected in 6-wells by the calcium phosphate method with 1 μg of a CD4, tetherin, NTB-A or CD1d expression vector and 5 μg of vectors co-expressing Vpu/Rev1-Vpu and eGFP or proviral HIV-1 M constructs. For experiments in primary cells, 1 million PHA-stimulated PBMCs were transduced with VSV-G-pseudotyped HIV-1. Two days post transfection (HEK293T cells) or three days post transduction (PBMCs), cells were stained extracellularly with antibodies against CD4 (monoclonal, Invitrogen, Cat# MHCD0405, dilution 1:40), tetherin (monoclonal, BioLegend, Cat# 348410, dilution 1:25), NTB-A (monoclonal, R&D, Cat# FAB19081A, dilution 1:25) or CD1d (monoclonal, BD, Cat# 550255, dilution 1:10). HEK293T cells transfected with proviral constructs and PBMCs were additionally permeabilized and stained for p24 (rabbit anti-p24 polyclonal rabbit antiserum, generated by Eurogentec; Alexa Fluor 488, life technologies, A11008, dilution 1:20). Fluorescence was detected by two-color flow cytometry and changes in tetherin, CD4, NTB-A and CD1d surface expression levels of eGFP or p24 positive cells were calculated.

### Virus release assay

To determine tetherin-mediated restriction of virion release HEK293T cells were seeded in 6-well plates and transfected with 5 μg of a proviral construct and increasing amounts of a plasmid coexpressing human tetherin and DsRed2. 40 h post transfection, cells and supernatants were lysed in Triton X-100 and the relative p24 release in the supernatant was determined by an home-made antigen enzyme-linked immunosorbent assay (ELISA).

### p24 ELISA

To quantify p24 amounts of HIV-1 M NL4-3, ZM246, and ZM247, a home-made sandwich ELISA was used. Briefly, 96-well plates were coated with an anti-p24 antibody from abcam (Cat# 9071, dilution 1:5,000), and bound p24 was detected using a polyclonal rabbit antiserum generated by Eurogentec (dilution 1:667) and an HRP-conjugated secondary antibody from Dianova (Cat# 111-035-008, dilution 1:2000). The protocol included several washing and blocking steps using Phosphate-Buffered Saline + 0.05% Tween (PBS-T) and PBS + 10% FCS, respectively.

### Particle infectivity assay

To determine virion infectivity, virus stocks from HEK293T cells were adjusted for their p24 content using a home-made p24 ELISA. Subsequently, TZM-bl reporter cells were infected with the adjusted virus stocks and β-galactosidase activity was measured three days post infection.

### NF-кB reporter assay

Transfections for the NF-кB luciferase reporter assay were performed in 96-well plates and each transfection was performed in triplicates. HEK293T cells were co-transfected with a firefly luciferase reporter construct under the control of three NF-кB binding sites (100 ng), a *Gaussia* luciferase construct under the control of a minimal pTAL promoter for normalization (25 ng), and a proviral HIV-1 construct (100 ng). Dual luciferase assays were performed 40 h post-transfection and the firefly luciferase signals were normalized to the internal *Gaussia* luciferase control.

### Rev reporter assay

The pNL-GFP-RRE(SA) reporter construct expresses GFP in a Tat- and Rev-dependent manner. In the absence of Rev, only fully spliced mRNA which lacks the GFP ORF is exported from the nucleus into the cytoplasm. HEK293T cells were co-transfected with 1 μg of pNL-GFP-RRE(SA), 1 μg of a pCG vector expressing BFP and increasing amounts of proviral HIV-1 M constructs (0.1 μg, 0.6 μg, 3.0 μg). Two days post transfection, GFP expression of BFP positive (*i*.*e*. transfected) cells was determined by flow cytometry.

### Viral replication in PBMCs and HLT

Virus stocks were generated by co-transfection of HEK293T cells with 5 μg of proviral DNA and 1 μg of pHIT/G. Two days post transfection, virus stocks were harvested and TZM-bl reporter cells were infected to adjust infectivity of the cell culture supernatants. Subsequently, PHA-stimulated PBMCs and HLT explants were infected with normalized virus stocks. HIV-1 replication was monitored by determining reverse transcriptase activity in the supernatants 0, 3, 5, 7 and 10 days post infection.

### Competition assay in PBMCs

1 million PHA-activated PBMCs were coinfected in 300 μl medium containing equal amounts of wild type and frameshift viruses encoding a *rev1-vpu* fusion gene or not. After 6 h, cells were washed three times and cultured in 2 ml of supplemented RPMI containing 10 ng/ml IL-2. Supernatants were collected 10 d after infection and RT-PCR (SuperScript III One-Step RT-PCR with Platinum Taq; Invitrogen) was performed to amplify viral genomic RNA using primers flanking the *rev1*-*vpu* intergenic region. The PCR fragments were purified from agarose gels and sequenced to determine the outcome of the competition.

### Reverse transcriptase assay

To determine reverse transcriptase activity, 6 μl of cell culture supernatant were added to 25 μl of a reverse transcription solution (50 mM Tris-HCl, 63 mM KCl, 4.2 mM MgCl_2_, 0.08% Nonidet P40, 1.68 mM EDTA, 4.2 mM polyA, 0.14 μg/ml oligo-dT, 4 mM DTT, 25 nCi ^32^P isotope) and incubated at 37°C for 2 h. Subsequently, 6 μl of the reaction mixture were transferred on a whatman filter paper and washed 3–5 times with 2x SSC buffer (300 mM NaCl, 30 mM Na_3_Citrate x2 H_2_O, pH 7.0). After washing with 96% ethanol, the filter was dried. Radioactivity was detected with a BAS 2000 Phospho-Imager and the signal was quantified using AIDA Image Analyzer.

### Analysis of the HIV-1 quasispecies in longitudinally studied patients

Previously published single genome sequences (SGS) from human subjects acutely infected with a single clade A or C transmitted founder virus and followed longitudinally for 1–2 years were retrieved from Genbank (for accession numbers see Table 1). Subjects were infected by heterosexual routes, identified at clinical sites in Rwanda, Malawi or South Africa, and remained treatment-naïve for the duration of study. These longitudinal sequences (half or near full length genomes) were originally generated to study virus sequence evolution in response to host immune responses [26]. Sequences were aligned, translated into the three forward reading frames, and evaluated for the presence of a *rev1-vpu* fusion gene.

### *In silico* analyses of RNA structure

mRNA structures were predicted using the Mfold web server for nucleic acid folding and hybridization prediction [37]. Linear r*ev/vpu/env* encoding mRNA spliced at donor 1 and acceptor 4c was used for modeling. 5’ and 3’ mRNA ends were not modified. *In silico* folding was performed at 37°C, 1M Na^+^ and 0 M Mg^++^. Both, the maximum size and maximum asymmetry of interior/bulge loops were set to 30. The maximum distance between paired bases was unlimited.

### Statistical analyses

Statistical calculations were performed with a two-tailed unpaired Student’s t test or one sample t test using Graph Pad Prism 5.03. P values <0.05 were considered statistically significant.
